# Evaluation of the Trauma and Injury Severity Score (TRISS) Performance in Intensive Care Trauma Patients: A Systematic Review and Meta-Analysis

**DOI:** 10.7759/cureus.95476

**Published:** 2025-10-26

**Authors:** Georgios Velonas, Dimitrios Mantas, Georgios Kassianidis, Vasiliki Poulianidou, Maria Patrani

**Affiliations:** 1 Critical Care Medicine, General Health Clinic Melathron TYPET, Athens, GRC; 2 Second Department of Propaedeutic Surgery, National and Kapodistrian University of Athens, Athens, GRC; 3 Critical Care Medicine, Korgialeneion-Benakeion Athens General Hospital, Athens, GRC

**Keywords:** critical care, injury severity score, intensive care units, mortality, multiple trauma

## Abstract

The Trauma and Injury Severity Score (TRISS) is a widely implemented tool for predicting outcomes in trauma patients. However, the application of the TRISS in intensive care units (ICUs) is limited, and data from international sources are scarce, as it requires adaptation to the demands of the specialized ICU environment. This article aimed to evaluate the predictive performance of the TRISS in critically ill adult patients with polytrauma admitted to ICU and to compare its prognostic accuracy against established ICU scoring systems. A comprehensive literature search was conducted across PubMed, Scopus, ScienceDirect, and CINAHL, in accordance with the Preferred Reporting Items for Systematic Reviews and Meta-Analysis (PRISMA) guidelines. The review included studies evaluating the predictive performance of TRISS in adult polytrauma patients admitted to the ICU. Two reviewers independently conducted study selection, data extraction, and risk of bias assessment. Cohen’s d values were synthesized via random-effects meta-analysis. Heterogeneity and publication bias were assessed using I^2 ^statistics and Egger’s test, respectively. The combined searches yielded 4,179 records, of which 24 studies were included in the systematic review and 21 in the meta-analysis. TRISS demonstrated a strong ability to discriminate between survivors and non-survivors, with a pooled effect size of Cohen’s d of -1.54 (95% CI: -1.73 to -1.35, p<0.001). In subgroup analyses based on equivalent study sets, Cohen’s d values were -1.55 for TRISS and 1.53 for Acute Physiology And Chronic Health Evaluation II (APACHE II), -1.20 for TRISS and 1.53 for APACHE III, and -1.71 for TRISS and 1.40 for Sequential Organ Failure Assessment (SOFA). Despite considerable heterogeneity among studies (I^2^>99%), no significant publication bias was detected. These findings indicate that TRISS has comparable prognostic accuracy to established ICU scoring systems. Additional clinical variables may further enhance predictive performance in critical care.

## Introduction and background

Globally, trauma is one of the leading causes of morbidity and mortality. According to the World Health Organization (WHO), both unintentional and violence-related injuries result in 4.4 million deaths per year and account for nearly 8% of all deaths worldwide. The main causes of death consist of injuries resulting from road traffic accidents, homicide, and suicide [1]. These traumatic conditions represent a serious public health issue, with socioeconomic and demographic dimensions as well as impacts on the human body (e.g., permanent disability) [2].

The term polytrauma patient has been widely used in international literature and refers to a patient who has suffered two or more major injuries [3]. However, according to the new Berlin definition, a polytrauma patient is defined as a patient who has an Abbreviated Injury Scale (AIS) ≥3 for two or more different body regions, with the addition of one or more of the following five physiological variables [4]. These variables consist of a systolic arterial pressure (SBP) of ≤90 mmHg, Glasgow Coma Scale (GCS) score ≤8, base excess ≤6.0, international normalized ratio (INR) ≥1.4, or partial thromboplastin time (PTT) ≥40 s, and age ≥70 years [4]. It is worth noting that the AIS itself has undergone several revisions over the years to improve its accuracy and clinical relevance, with the most recent version being AIS 2015 [5].

These patients often require intensive care and multilevel treatment. Studies have shown that multiple trauma patients account for a high proportion of admissions to intensive care units (ICUs), while common complications, such as infections, acute renal failure, and acute respiratory distress syndrome (ARDS), are associated with increased mortality, morbidity, and length of stay [6,7].

Various scoring scales have been developed to categorize and predict the course and outcome of patients with multiple traumatic injuries. Specifically, the Injury Severity Score (ISS) is calculated as the sum of squares of the three most severe injuries in different body regions based on the AIS, while the Revised Trauma Score (RTS) incorporates the GCS, SBP, and respiratory rate (RR) [8]. These scales help to assess trauma and estimate the likelihood of survival in order to guide the treatment plan of care.

The Trauma and Injury Severity Score (TRISS) is one of the most comprehensive and widely used scales in patients with multiple trauma. The coefficients of the current TRISS model were calculated based on the dataset from the Major Trauma Outcome Study (MTOS) coordinated by the American College of Surgeons Committee on Trauma [9]. It is a weighted combination of ISS, RTS, and the age of the injured patient, which produces a score ranging from 0 to 1 and can be interpreted as the estimated probability of survival [8,9]. This scale also provides reliable predictions in patients with severe trauma, and its accuracy has been improved by incorporating modern analyses and new parameters [10,11].

In contrast to other scales, such as the Acute Physiology and Chronic Health Evaluation II (APACHE II) and the Sequential Organ Failure Assessment (SOFA), which are based primarily on clinical data of patients admitted to the ICU, the TRISS scale potentially provides a more comprehensive picture of trauma severity, taking into account the anatomical features of the polytrauma patient [12,13]. However, the application of the TRISS scale in ICUs is limited, and data from international sources are scarce, as it requires adaptation to the demands of the specialized ICU environment. Given that ICU populations represent a subgroup with distinct clinical complexity and higher severity of illness, there is a need to comprehensively assess their performance in this specific setting. In the present study, a systematic review and meta-analysis were conducted to evaluate the effectiveness of the TRISS scale as a tool to estimate survival in adult multitrauma patients admitted to the ICU.

## Review

Methods

Reporting Standards

This systematic review and meta-analysis were conducted in accordance with the Preferred Reporting Items for Systematic Reviews and Meta-Analyses (PRISMA) guidelines [14].

Eligibility Criteria

Eligible studies were those evaluating the Trauma and Injury Severity Score (TRISS) as a prognostic tool for outcome prediction in adult polytrauma patients admitted to the ICU [15]. Both prospective and retrospective observational studies were included, provided they assessed the predictive performance of TRISS, with or without comparison to other prognostic scoring systems, specifically the APACHE II, APACHE III, and SOFA scores [16-18].

Studies were excluded if TRISS was applied in non-ICU environments (e.g., prehospital care, emergency departments, general wards), if they focused solely on isolated trauma types (e.g., traumatic brain injury), or lacked primary data (e.g., reviews, case reports, conference abstracts). Only studies published in English were included. No publication year restrictions were applied, as TRISS was originally introduced in 1981; thus, all studies since its development were considered relevant to capture its full clinical evolution [15].

Information Sources and Search Strategy

The literature search was conducted using four electronic databases as follows: PubMed, Scopus, ScienceDirect, and CINAHL. The final search was completed on May 1, 2025. The search strategy employed a sequential approach to capture all relevant studies on TRISS, including those using it in isolation and those comparing it with other scoring systems. The search terms included "TRISS," "Trauma and Injury Severity Score," "Intensive Care Units," "ICU," "critical care," "multiple trauma," "trauma," "severe injury," "APACHE II," "APACHE III," and "SOFA." Boolean operators and database-specific subject headings (e.g., MeSH) were applied. No filters for publication date or study design were applied. Searches were performed directly within the database search engines. The full electronic search strategy for each database is provided in the table in appendix.

Selection Process

Two independent reviewers conducted the selection process. Prior to screening, duplicate records retrieved across databases were identified and removed. Initially, titles and abstracts were screened to exclude irrelevant studies, followed by a full-text review of potentially eligible articles. First, studies not evaluating the TRISS score were excluded. Then, studies applying TRISS outside the ICU setting were removed. Finally, studies focusing solely on specific organ trauma or subgroups were excluded. Discrepancies between reviewers were resolved through consensus meetings. Although all eligible studies were included in the qualitative synthesis, some were excluded from the meta-analysis due to reasons such as lack of extractable data, use of incompatible outcome measures, or significant methodological differences that prevented data pooling. Reasons for exclusion from the meta-analysis are detailed in the results section.

Data Collection Process and Data Items

Data extraction was performed independently by two reviewers using structured forms. Regular meetings between reviewers ensured cross-checking and consistency. No contact was made with the original study authors to request additional information. All prognostic scoring systems evaluated in this study are widely used in clinical practice and research, publicly available, and free to use.

The following extracted items were included: author, year of publication, study type, number of patients, number of hospitals, country, prognostic scores evaluated, area under the receiver operating characteristic curve (AUROC), and results. In addition, data regarding patient outcomes (survivors versus non-survivors) were collected to enable subsequent meta-analytical synthesis.

The outcomes of interest included survival and mortality rates among ICU polytrauma patients as predicted by the TRISS score. AUROC values were extracted when available to assess discriminative ability. Comparative performance between TRISS and other prognostic scores (APACHE II, APACHE III, SOFA) was evaluated. Given that the TRISS score predicts survival probability [15], while the APACHE and SOFA scores predict mortality, appropriate harmonization of the outcome direction was considered during data extraction and analysis [16-18]. No assumptions were made regarding missing or ambiguous data; only explicitly reported values were considered.

Study Risk of Bias Assessment

Risk of bias was independently assessed by two reviewers using the Quality in Prognostic Studies (QUIPS) tool [19]. The following six domains were evaluated: study participation, attrition, prognostic factor measurement, outcome measurement, confounding and statistical analysis, and reporting. Each domain was graded as low, moderate, or high risk of bias. Regular meetings were held to resolve any disagreements. No automation tools were used in this process.

Statistical Analysis

Statistical analysis was performed using SPSS version 29.0 (Armonk, NY: IBM Corp). All data that were retrieved from eligible studies were transformed into Cohen’s d value. Transformation of the data with area under the curve (AUC) into Cohen’s d value was achieved using the procedure described in the study by Salgado [20].

The TRISS between the group of survivors (controls) and the non-survivors group (deceased) was calculated using Cohen’s d value (standardized mean difference {SMD}) with a 95% confidence interval (CI). The significance of the pooled SMD was determined by the Z-test. A random-effect model was applied respectively for heterogeneous data after calculating Cochran’s Q-statistic (p<0.05 was significant) and I^2^ test (0%, no heterogeneity; 100%, maximal heterogeneity). A funnel plot and Egger’s test were used to estimate the publication bias. The statistical significance level was set at 5% (p<0.05).

A subgroup analysis was performed based on the prognostic score evaluated (TRISS, APACHE II, APACHE III, SOFA) to assess differences in predictive performance between models. No sensitivity analysis was conducted. No formal certainty assessment (e.g., Grading of Recommendations Assessment, Development, and Evaluation {GRADE}) was conducted, as the review was based on observational studies of prognostic accuracy.

Results

Study Selection

**Figure 1:**
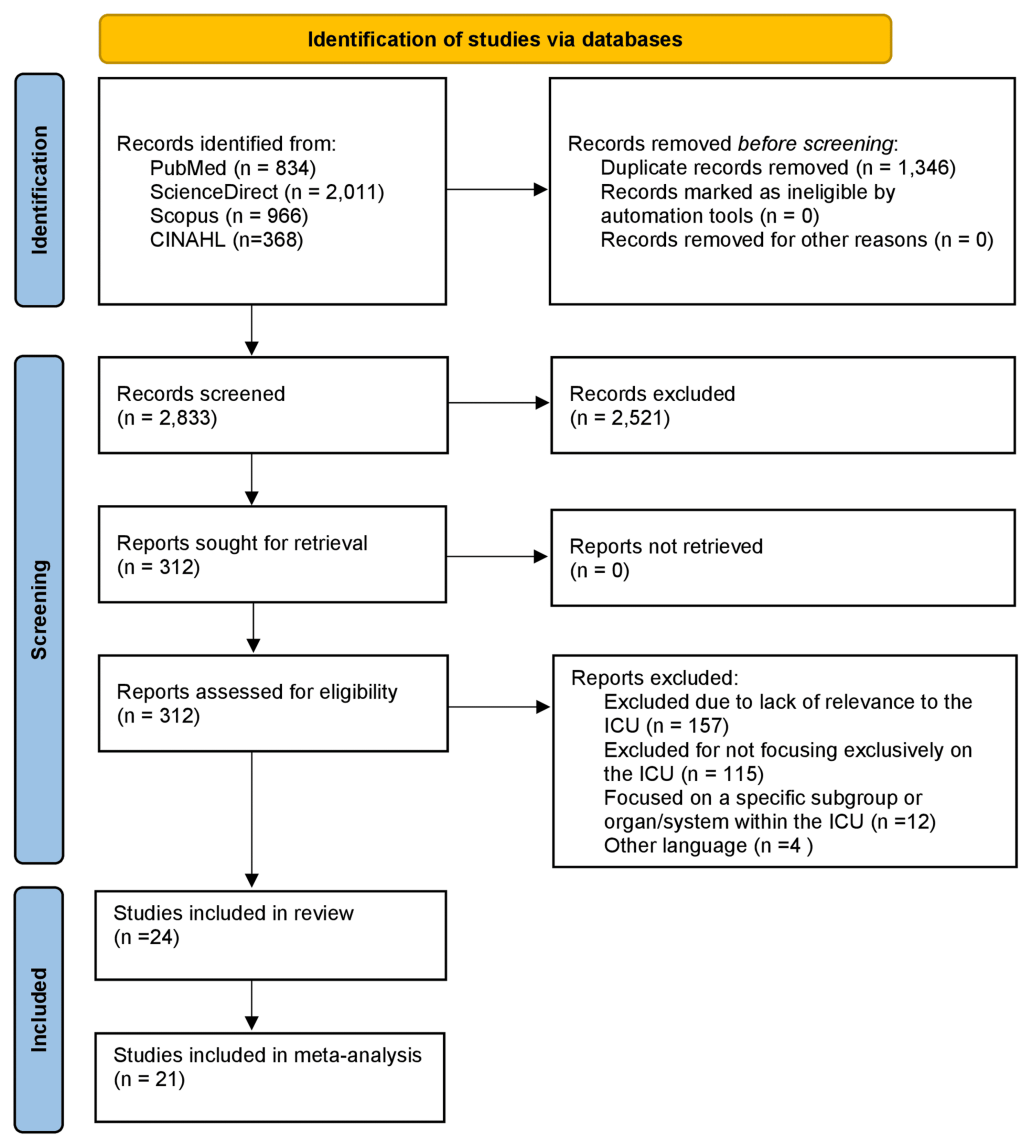
PRISMA flow diagram of study selection. A flow diagram representing the study selection process according to PRISMA guidelines. The figure shows the number of records identified, screened, excluded, and included in the final review. PRISMA: Preferred Reporting Items for Systematic Reviews and Meta-Analyses

A total of 4,179 records were identified through database searches. After removing 1,346 duplicate records, 2,833 records remained for title and abstract screening. During this screening phase, 2,521 records were excluded for featuring pediatric populations, lacking a focus on ICU trauma patients, or not including an evaluation of TRISS. The remaining 312 reports were assessed in full-text format for eligibility. No reports were excluded due to retrieval issues. Among them, we excluded 288 articles by applying our inclusion and exclusion criteria. Ultimately, 24 studies met the eligibility criteria and were included in the systematic review [21-44]. Of these, 21 studies were included in the meta-analysis, while three studies were excluded from the meta-analysis due to a lack of extractable quantitative data or incompatible outcome reporting [21-26,28-30,32,33,35-44]. More specifically, the study by Wu et al. was excluded from the meta-analysis, as the authors applied a transformation to the data prior to presentation [34]. Specifically, the TRISS score was calculated based on a logarithmic regression model, rendering the results incompatible with the inclusion criteria. Similarly, the study by Manikis et al. was excluded due to the lack of suitably reported data [27], while the study by Reiter et al. was excluded because not all required data were reported [31]. The PRISMA 2020 flow diagram illustrating the study selection process is provided in Figure 1 [14].

Study Characteristics

A total of 24 studies were included in the systematic review, published between 1993 and 2023 [21-44]. Study designs were predominantly retrospective observational cohorts, with some prospective studies. The included studies originated from a wide variety of countries, reflecting different healthcare systems and ICU settings. Sample size ranged from 56 to 5,538 patients.

All studies focused on evaluating the prognostic accuracy of the TRISS in predicting outcomes among adult polytrauma patients admitted to the ICU [21-44]. While some studies exclusively assessed the TRISS score [27,33,44], others compared its predictive performance with additional scoring systems [21-26,28-32,34-43]. Patient outcomes of interest included survival and mortality rates during ICU admission. The extracted data also encompassed AUROC values and the number of survivors and non-survivors. Detailed characteristics of the included studies are summarized in Table 1.

**Table 1:** Main characteristics of the included studies. ANZROD: Australian and New Zealand Risk of Death; APACHE II/III: Acute Physiology and Chronic Health Evaluation II/III; ARDS: acute respiratory distress syndrome; AUROC: area under the receiver operating characteristic curve; BISS: Base Excess Injury Severity Scale; BNISS: Base Excess New Injury Severity Score; CI: confidence interval; D-SOFA: delta SOFA (total maximum SOFA score minus admission total SOFA); GAP: Glasgow Coma Scale, age, and systolic blood pressure; GCS: Glasgow Coma Scale; HRV: heart rate variability; ICU: intensive care unit; ISS: Injury Severity Score; KTS: Kampala Trauma Score; LODS: Logistic Organ Dysfunction Score; MGAP: mechanism, GCS, age, and systolic blood pressure; MODS: Multiple Organ Dysfunction Score; MOF: multiple organ failure; MPM II: mortality probability model II; mREMS: modified Rapid Emergency Medicine Score; nLF/nHF: normalized low frequency/high frequency ratio; HRV: heart rate variability; NISS: New Injury Severity Score; NR: not reported; NRS-2002: Nutritional Risk Screening 2002; NTRISS: New Trauma and Injury Severity Score; PPV: positive predictive value; PS14: probability of survival 2014; Ps (TRISS): probability of survival by Trauma and Injury Severity Score; PTS: Polytrauma Score; RISC II: Revised Injury Severity Classification II; ROD: risk of death; RTS: Revised Trauma Score; SAPS II: Simplified Acute Physiology Score II; SOFA: Sequential Organ Failure Assessment; SSS: Sepsis Severity Score; T-RTS: Triage Revised Trauma Score; TMS: total maximum SOFA (maximum SOFA score during ICU stay); TRIOS: Three Days Recalibrated ICU Outcome Score; TRISS: Trauma and Injury Severity Score; TS: Trauma Score

Studies	Type of study	Number of patients (n)	Number of hospitals (N)	Country	Prognostic scores	AUROC	Findings
Wong et al. (1996) [21]	Prospective study	470	1	Canada	APACHE II, TRISS	APACHE II: 0.92±0.02, TRISS: 0.89±0.02	Both APACHE II and TRISS scores were shown to accurately predict group mortality in ICU trauma patients. However, APACHE II tended to underestimate actual death rates, especially at high-risk ranges, while TRISS tended to overestimate them. Neither system was found to be satisfactory in predicting individual outcomes.
Thanapaisal and Saksaen (2012) [22]	Prospective study	132	1	Thailand	APACHE II, TRISS	APACHE II: 0.89±0.04, TRISS: 0.83±0.04	Both APACHE II and TRISS scores were shown to accurately predict group mortality in ICU trauma patients. However, predicted death rates were overestimated actual observed death rates. Neither APACHE II nor TRISS provides sufficient confidence for prediction of outcome of individual patients.
Mazandarani et al. (2016) [23]	Prospective cross-sectional study	152	2	Iran	APACHE III, TRISS	TRISS: 0.806, APACHE III: 0.797	TRISS and APACHE models have the same accuracy in predicting mortality of ICU-admitted trauma patients, yet the TRISS model would be more applicable because of its easier calculation, consideration of trauma characteristics, and independence from patient care quality.
Kroezen et al. (2007) [24]	Prospective study	528	2	The Netherlands	TRISS, BISS	Tilburg: TRISS 0.806, BISS 0.803 Utrecht: TRISS 0.891, BISS 0.885	The performance of BISS was superior to that of the TRISS model in the studied populations, while delta base deficit is significantly correlated with mortality.
Magee et al. (2021) [25]	Prospective multicentre study	5,237	25	Australia	ANZROD, APACHE III, APACHE III ROD, TRISS, ISS, NISS, RTS	ANZROD: 0.91, APACHE III: 0.88, APACHE III ROD: 0.88, TRISS: 0.78, ISS: 0.61, NISS: 0.68, RTS: 0.69	The performance of each scoring system was highest in younger adults and poorest in older adults, whereas ANZROD and APACHE III had a superior performance when compared with traditional trauma-specific scoring systems.
Hwang et al. (2012) [26]	Retrospective cohort study	706	1	South Korea	SOFA, APACHE II, TRISS	SOFA: 0.953, APACHE II: 0.950, TRISS: 0.922	SOFA was not different from APACHE II and TRISS in predicting the outcomes for ICU trauma patients, yet SOFAs calculation is easier and simpler.
Manikis et al. (1995) [27]	Prospective study	129	1	Belgium	TRISS, Serial arterial blood lactate	NR	TRISS correlated well with the development of organ failure, and serial blood lactate measurements are reliable indicators of morbidity and mortality after trauma. Moreover, not only the initial or the highest lactate value but also the duration of hyperlactatemia can be correlated with the development of organ failure.
Agarwal et al. (2015) [28]	Retrospective study	95	1	India	APACHE II, TRISS	APACHE II day 1: 0.885, TRISS: 0.831, APACHE II (admission): 0.706	APACHE II score on day one of admission was a relatively better predictor than TRISS, although it underestimated mortality, while specificity for TRISS was better than that of APACHE II score on day one.
Cabrejas et al. (2020) [29]	Retrospective observational study	1,240	1	Spain	TRISS, TRISS 2010, PS14	TRISS: 0.915, TRISS 2010: 0.919, PS14: 0.914	Higher than expected survival rates were observed, with no significant differences in discrimination among models. These models are suitable tools for assessing quality of care in a trauma ICU.
Corbanese et al. (1998) [30]	Prospective study	162 TRISS, 108 APACHE II	1	Italy	APACHE II, TRISS	TRISS: 0.963, APACHE II: 0.902±0.066	TRISS performed slightly better than APACHE II score as an audit system, showing larger positive and smaller negative likelihood ratios. APACHE II was probably influenced by the low number of cases.
Reiter et al. (2004) [31]	Prospective multicenter cohort study	5,538	31	Austria	SAPS II, TRISS, TRISS-SAPS	TRISS: 0.84, SAPS II: 0.87, TRISS-SAPS: 0.89	TRISS underestimates mortality, especially in patients over 60 years, while SAPS II overestimates mortality, pronounced in young patients. Combining TRISS and SAPS II significantly improved both calibration and discrimination.
Köseoğlu et al. (2011) [32]	Prospective study	100	1	Turkey	APACHE II, TRISS, ISS, NRS-2002	TRISS: 0.926, APACHE II: 0.920, ISS: 0.878, NRS-2002 (complications): 0.708	ISS, TRISS, and APACHE II at admission had a reliable power of discrimination for mortality and complication prediction. The NRS-2002 score was significantly increased in patients with complications, but not associated with mortality.
Chico-Fernández et al. (2016) [33]	Prospective multicenter study	1,405	13	Spain	TRISS	Overall: 0.889, blunt: 0.887, penetrating: 0.919	TRISS underestimated mortality when the predicted mortality was below 60% and overestimated it when the predicted mortality was over 60%, showing inadequate calibration, especially in blunt trauma. Penetrating trauma showed better discrimination and good calibration.
Wu et al. (2020) [34]	Prospective study	1,554	1	Taiwan	TRISS, APACHE II, SAPS II, MPM II (admission, 24 h, 48 h, 72 h), MODS, SOFA, LODS, TRIOS	MPM II 24 h: 0.9213, MPM II 48 h: 0.9105, MPM II admission: 0.9063, SAPS II: 0.9044, LODS: 0.9013, APACHE II: 0.8923, TRISS: 0.8814, TRIOS: 0.8701, MODS: 0.8179, SOFA: 0.7073	MPM II at 24 h had the best predictive performance, and SOFA does not predict death as well when it uses exact numbers instead of simple scores from 0 to 4. TRISS becomes less accurate when it does not use lab test results.
Rio et al. (2023) [35]	Retrospective cohort study	747 (106 ICU patients)	1	Brazil	RTS, NTS, mREMS, ISS, NISS, TRISS, NTRISS, BISS, BNISS	BNISS: 0.976, NTRISS: 0.967, TRISS: 0.909, BISS: 0.902	ISS and NISS had better predictive capacity for patient admission to the ICU, while NISS, TRISS, NTRISS, BISS, and BNISS showed excellent performance in predicting mortality in the ICU. TRISS was the one with the highest accuracy (96.2%) and PPV (66.7%).
Mijaljica et al. (2022) [36]	Prospective study	75	1	Serbia	ISS, NISS, APACHE II, SOFA, TRISS, KTS	TRISS: 0.900, APACHE II: 0.866, ISS: 0.860, NISS: 0.853, KTS: 0.849, SOFA: 0.836	All trauma scores were significant predictors of mortality, with TRISS and APACHE II emerging as the most powerful mortality predictors. KTS did not perform as expected.
Vassar et al. (1999) [37]	Retrospective multicenter study	2,414	6	United States	APACHE II, APACHE III, TRISS, 24-hour ICU points	APACHE II: 0.87, APACHE III: 0.89, TRISS: 0.82, 24-h ICU points: 0.89	APACHE III and 24-h ICU point system demonstrated excellent calibration and discrimination, whereas APACHE II and TRISS were poor predictors of aggregate mortality. In patients without head injuries, all systems were unreliable and considerably underestimated the risk of death.
Papadimitriou-Olivgeris et al. (2021) [38]	Retrospective study	326	1	Greece	ISS, NISS, RTS, TRISS, RISC II, APACHE II, SAPS II, SOFA, GCS	NISS: 0.901, RISC II: 0.883, TRISS: 0.859, ISS: 0.820, RTS: 0.742, APACHE II: 0.698, SAPS II: 0.670, SOFA: 0.690, GCS: 0.691	Multivariate analysis showed that higher NISS, severe head or neck injury, acute kidney injury, septic shock, and hemorrhagic shock were significantly associated with mortality, while RISC II and the administration of enteral nutrition were associated with survival. NISS showed higher accuracy in predicting 30-day mortality followed by RISC II.
Fueglistaler et al. (2010) [39]	Prospective study	237 (211 ICU patients), TRISS (n=147)	1	Switzerland	SAPS II, SOFA (day 1, TMS, D-SOFA), ISS, RTS, PTS, TRISS, SAPS+SOFA, TRISS+SOFA	SAPS II: 0.86, TRISS: 0.83, SOFA day 1: 0.79, ISS: 0.80, SAPS+SOFA: 0.87, TRISS+SOFA: 0.86	SAPS II yielded the best predictive value for mortality, while SOFA score significantly added prognostic information about mortality to both SAPS II and TRISS. Adding SOFA to SAPS II, PTS, or TRISS, as well as PTS or TRISS to SAPS II, significantly increased the accuracy of mortality prediction.
Dossett et al. (2009) [40]	Prospective cohort study	1,019	2	United States	APACHE II, ISS, TRISS	APACHE II: 0.77, TRISS: 0.64, ISS: 0.54, APACHE II (penetrating): 0.81, APACHE II (blunt): 0.76	APACHE II was superior in predicting mortality, with GCS, temperature, and serum chemistries contributing most to APACHE II’s predictive ability. TRISS underestimated mortality in low-risk patients and overestimated it in high-risk patients.
Llompart-Pou et al. (2017) [41]	Prospective multicenter study	1,361	34	Spain	TRISS, MGAP, GAP, T-RTS	TRISS: 0.897, MGAP: 0.860, GAP: 0.849, T-RTS: 0.796	MGAP and GAP scores performed better than the T-RTS in predicting mortality. The TRISS model slightly underestimated overall mortality compared to the observed rate.
Roumen et al. (1993) [42]	Prospective cohort study	56	3	Austria, Netherlands	ISS, TRISS, TS, PTS, GCS, APACHE II, SSS	NR	Injury severity scoring systems positively correlate with subsequent ARDS and MOF. APACHE II, although significantly different between survivors and non-survivors, had no correlation with subsequent ARDS or MOF.
Serviá et al. (2019) [43]	Prospective cohort study	780	1	Spain	ISS, NISS, RTS, APACHE II, MPM II-24, TRISS	APACHE II: 0.87, ISS: 0.73, NISS: 0.78, RTS: 0.76, MPM II: 0.83, TRISS: 0.80, APACHE II+ISS: 0.88	Combining APACHE II with ISS yielded the best predictive model. Anatomical scores like ISS and NISS do not show a linear increase in mortality; their predictive reliability drops particularly in low-score patients. The TRISS score demonstrated good discriminatory ability, significantly distinguishing survivors from non-survivors, but showed poor calibration.
Luo et al. (2021) [44]	Prospective study	210	1	China	nLF/nHF (HRV), TRISS, RTS, NISS, ISS	30-day mortality: nLF/nHF: 0.924, RTS: 0.951, Ps (TRISS): 0.892, nLF/nHF+RTS: 0.979, nLF/nHF+TRISS: 0.984, MODS: nLF/nHF: 0.826, NISS: 0.818, RTS: 0.850, nLF/nHF+RTS: 0.884, nLF/nHF+NISS: 0.868	The nLF/nHF ratio, RTS, and Ps (TRISS) were found to be independent predictors of 30-day mortality. The spectral HRV index nLF/nHF showed comparable discriminatory accuracy to RTS and Ps (TRISS) for predicting 30-day mortality. Combining nLF/nHF with RTS or with Ps (TRISS) significantly enhanced predictive accuracy compared to using any of them alone.

Risk of Bias in Studies

**Figure 2:**
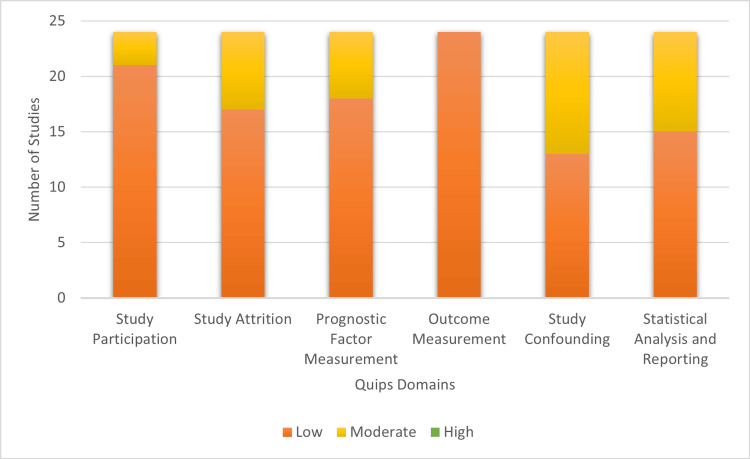
Summary of the risk of bias assessment using the QUIPS tool. Illustration created by the authors based on Quality in Prognostic Studies (QUIPS) tool [19]. Data synthesized from included studies [21-44].

The risk of bias was independently assessed for all included studies using the Quality in Prognostic Studies (QUIPS) tool [19]. Overall, the methodological quality of the included studies was moderate to good [21-44]. The domains of study participation and outcome measurement were consistently rated as low risk of bias across the majority of studies. However, moderate risk of bias was more frequently observed in the domains of prognostic factor measurement and study confounding, although the underlying reasons varied across studies (e.g., retrospective data collection or restricted adjustment due to study populations without major comorbidities). Only a small number of studies exhibited issues related to statistical analysis and reporting, and no domain was predominantly marked by a high risk of bias [23,27,32,35,38,40,41,43,44]. Regular meetings between reviewers ensured consensus was reached for all assessments (Table 2). A detailed summary of the risk of bias assessments is provided in Figure 2.

**Table 2:** Risk of bias of included studies according to the QUIPS tool. Assessment based on the criteria of the Quality in Prognostic Studies (QUIPS) tool [19].

Studies	Study participation	Study attrition	Prognostic factor measurement	Outcome measurement	Study confounding	Statistical analysis and reporting
Wong et al. (1996) [21]	Low	Low	Moderate	Low	Moderate	Low
Thanapaisal and Saksaen (2012) [22]	Low	Low	Moderate	Low	Moderate	Low
Mazandarani et al. (2016) [23]	Low	Low	Moderate	Low	Moderate	Moderate
Kroezen et al. (2007) [24]	Low	Low	Low	Low	Low	Low
Magee et al. (2021) [25]	Low	Moderate	Low	Low	Low	Low
Hwang et al. (2012) [26]	Low	Moderate	Low	Low	Low	Low
Manikis et al. (1995) [27]	Low	Moderate	Moderate	Low	Moderate	Moderate
Agarwal et al. (2015) [28]	Moderate	Moderate	Low	Low	Moderate	Low
Cabrejas et al. (2020) [29]	Low	Low	Low	Low	Low	Low
Corbanese et al. (1998) [30]	Moderate	Moderate	Low	Low	Moderate	Low
Reiter et al. (2004) [31]	Low	Low	Low	Low	Low	Low
Köseoğlu et al. (2011) [32]	Low	Low	Moderate	Low	Moderate	Moderate
Chico-Fernández et al. (2016) [33]	Low	Moderate	Low	Low	Moderate	Low
Wu et al. (2020) [34]	Low	Low	Low	Low	Moderate	Low
Rio et al. (2023) [35]	Low	Low	Low	Low	Moderate	Moderate
Mijaljica et al. (2022) [36]	Low	Low	Low	Low	Low	Low
Vassar et al. (1999) [37]	Low	Low	Low	Low	Low	Low
Papadimitriou-Olivgeris et al. (2021) [38]	Low	Low	Low	Low	Low	Moderate
Fueglistaler et al. (2010) [39]	Low	Low	Low	Low	Low	Low
Dossett et al. (2009) [40]	Low	Low	Moderate	Low	Low	Moderate
Llompart-Pou et al. (2017) [41]	Moderate	Moderate	Low	Low	Moderate	Moderate
Roumen et al. (1993) [42]	Low	Low	Low	Low	Low	Low
Serviá et al. (2019) [43]	Low	Low	Low	Low	Low	Moderate
Luo et al. (2021) [44]	Low	Low	Low	Low	Low	Moderate

Predictive Ability of TRISS in ICU Polytrauma Patients

Most studies demonstrated moderate to excellent discriminative ability of TRISS, with AUROC values commonly ranging from 0.64 to 0.96 [21-26,28-41,43,44]. Specifically, Köseoğlu et al. and Mijaljica et al. pointed out that TRISS was the most accurate mortality predictor among the systems tested, with AUCs of 0.926 and 0.900, respectively [32,36]. Similarly, Cabrejas et al. and Corbanese et al. confirmed TRISS's excellent performance, reporting AUC values of 0.91 and 0.96 [29,30].

However, several studies have outlined limitations. Magee et al. reported moderate accuracy for TRISS (AUC 0.78), inferior to ICU-based scores, such as Australian and New Zealand Risk of Death (ANZROD) (AUC 0.91) and APACHE III (AUC 0.88) [25]. TRISS’s performance notably declined in elderly patients, with an AUC of 0.64 [25], a finding similarly noted by Reiter et al., who found TRISS tended to underestimate mortality in patients over 60 years of age [31]. In terms of trauma types, TRISS was more accurate for predicting mortality in cases of penetrating trauma compared to blunt trauma [33]. Also, it tended to overestimate mortality in mild trauma [26].

Results comparing TRISS with other prognostic systems yielded mixed outcomes. ICU trauma studies often found that APACHE II and Simplified Acute Physiology Score II (SAPS II) outperformed TRISS, especially in discrimination and calibration [34,40,43]. ISS and New Injury Severity Score (NISS) showed better predictive power for ICU admission and mortality, with NISS consistently outperforming TRISS in multiple studies [35,38]. Newer combined scoring systems like Revised Injury Severity Classification II (RISC II), New Trauma and Injury Severity Score (NTRISS), and Base Excess Injury Severity Scale (BISS) sometimes matched or exceeded TRISS’s performance [24,35].

Furthermore, integrating TRISS with additional clinical variables, such as heart rate variability indices (e.g., normalized low frequency/high frequency {nLF/nHF} ratio) [44], base deficit measurements [24], or other clinical scores, significantly improved mortality predictions in various studies [31,39]. In terms of predicting complications such as ARDS or multiple organ failure (MOF), however, TRISS proved inadequate. Roumen et al. found that while TRISS predicted mortality effectively, it wasn’t reliable for forecasting later complications [42].

TRISS remains a valuable tool for group outcome prediction and quality assurance [22]. However, its accuracy for predicting individual patient outcomes, particularly in ICU settings and among patients with major comorbidities, appears limited [21,25,37].

Meta-Analysis

**Figure 3:**
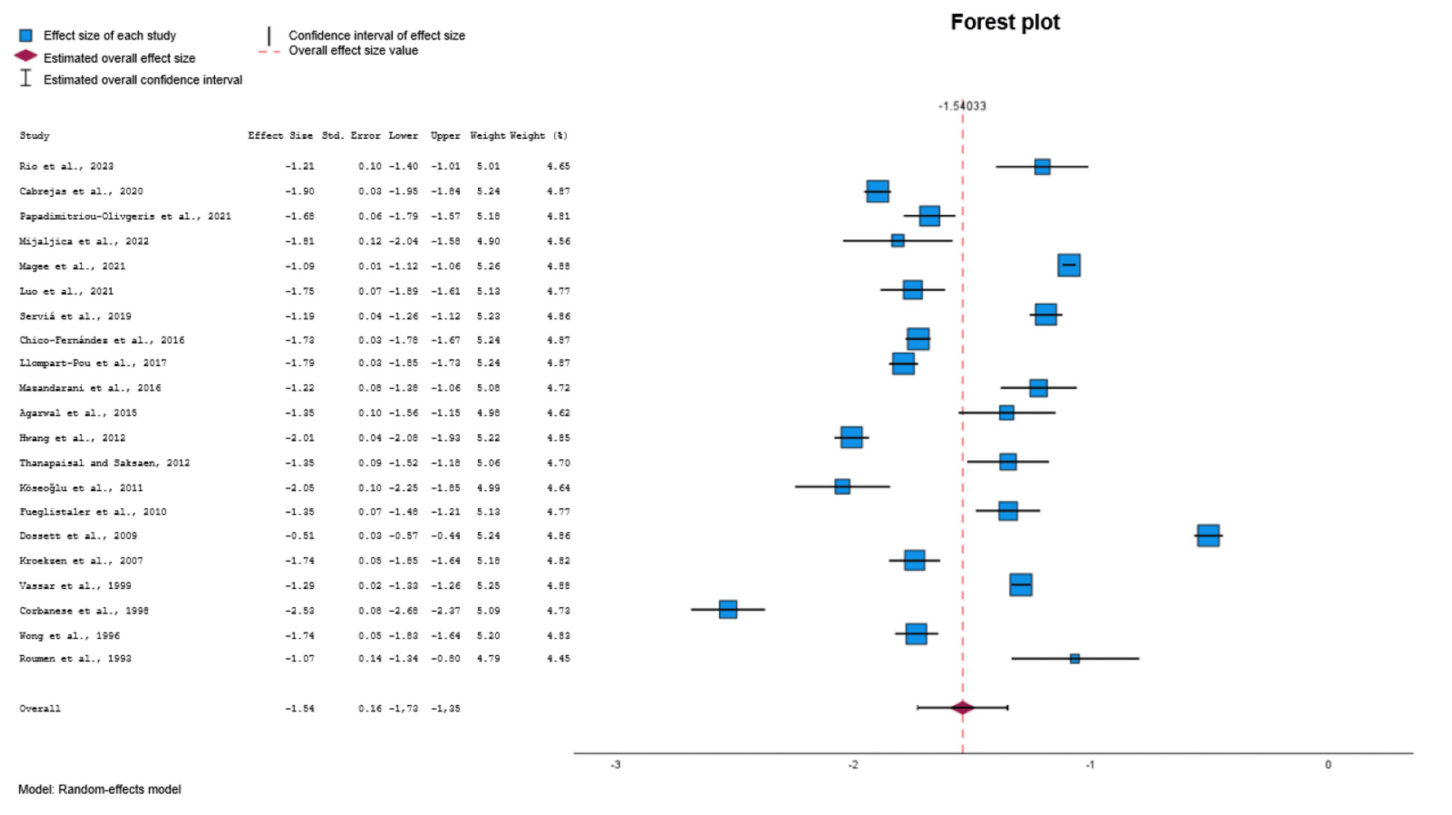
Forest plot comparing TRISS values between survivors and non-survivors. Data synthesized from included studies [21-26,28-30,32,33,35-44]. TRISS: Trauma and Injury Severity Score

Among the eligible studies [21-44], 21 were included in the meta-analysis for the TRISS (Figure 3) [21-26,28-30,32,33,35-44]. After conducting a random effects model, a moderate estimate of effect size (Cohen’s d value = -1.54, 95% CI: -1.73 to -1.35, p<0.001) was revealed, indicating that the TRISS was significantly different in the group of survivors (controls) compared to non-survivors group (deceased). Significant heterogeneity was identified across included studies (p<0.001, I^2^=99.2%). Visual examination of funnel plots and Egger's test (p=0.808) indicated that no significant publication bias was revealed over all included studies.

**Figure 4:**
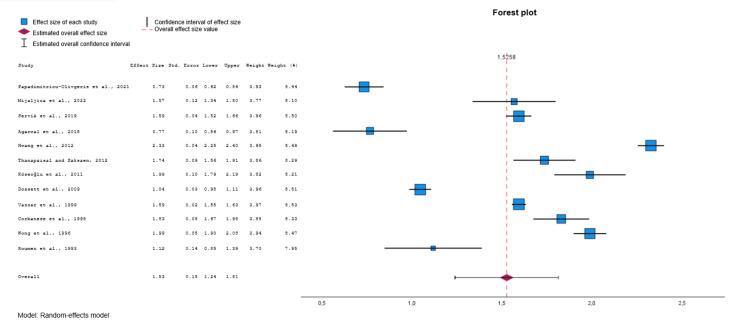
Forest plot comparing APACHE II values between survivors and non-survivors. Data synthesized from included studies [21,22,26,28,30,32,36-38,40,42,43]. APACHE II: Acute Physiology and Chronic Health Evaluation II

**Figure 5:**
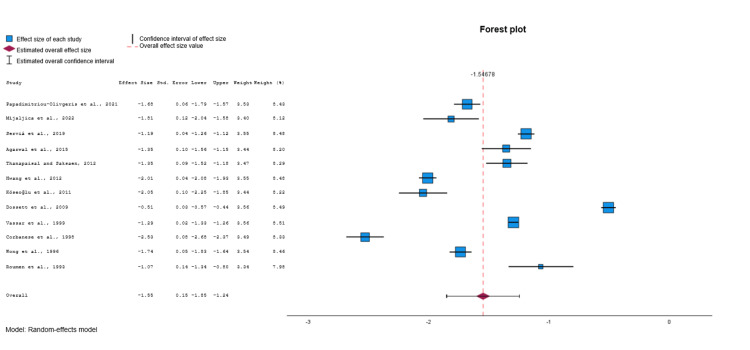
Forest plot comparing TRISS values (subgroup of 12 studies) between survivors and non-survivors. Data synthesized from included studies [21,22,26,28,30,32,36-38,40,42,43]. TRISS: Trauma and Injury Severity Score

Among the eligible studies [21-44], 12 were included in the meta-analysis for the APACHE II (Figure 4) [21,22,26,28,30,32,36-38,40,42,43]. After conducting a random effects model, a moderate estimate of effect size (Cohen’s d value=1.53, 95% CI: 1.24 to 1.81, p<0.001) was revealed, indicating that the APACHE II was significantly different in the group of survivors (controls) compared to the non-survivors group (deceased). Significant heterogeneity was identified across included studies (p<0.001, I^2^=99.1%). Visual examination of funnel plots and Egger's test (p=0.543) indicated that no significant publication bias was revealed over all included studies. For these 12 studies, the moderate estimate of effect size of TRISS was approximately equal to before (Cohen’s d value = -1.55, 95% CI: -1.85 to -1.24, p<0.001) (Figure 5) [21,22,26,28,30,32,36-38,40,42,43].

**Figure 6:**
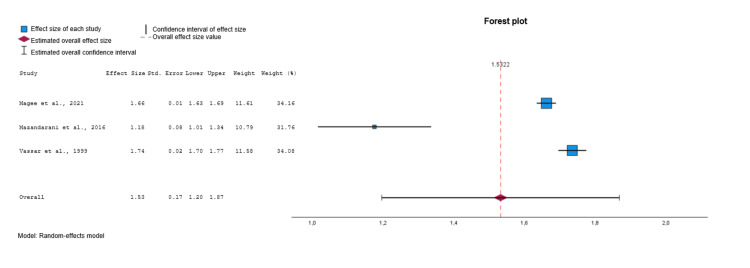
Forest plot comparing APACHE III values between survivors and non-survivors. Data synthesized from included studies [23,25,37]. APACHE III: Acute Physiology and Chronic Health Evaluation III

Among the eligible studies [21-44], three were included in the meta-analysis for the APACHE III (Figure 6) [23,25,37]. After conducting a random effects model, a moderate estimate of effect size (Cohen’s d value=1.53, 95% CI: 1.20 to 1.87, p<0.001) was revealed, indicating that the APACHE III was significantly different in the group of survivors (controls) compared to the non-survivors group (deceased). Significant heterogeneity was identified across included studies (p<0.001, I^2^=99.3%). Visual examination of funnel plots and Egger's test (p=0.164) indicated that no significant publication bias was revealed over all included studies. For these three studies, the moderate estimate of effect size of TRISS was higher than before (Cohen’s d value = -1.20, 95% CI: -1.33 to -1.07, p<0.001) [23,25,37].

**Figure 7:**
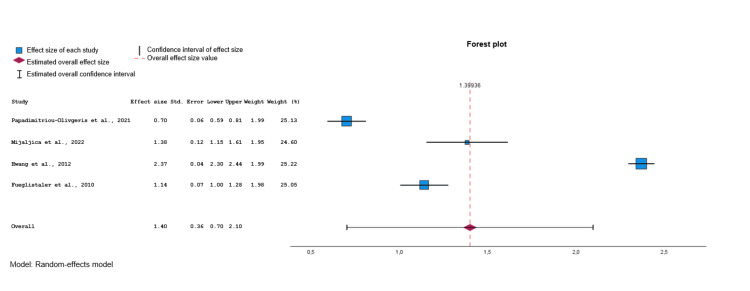
Forest plot comparing SOFA values between survivors and non-survivors. Data synthesized from included studies [26,36,38,39]. SOFA: Sequential Organ Failure Assessment

Among the eligible studies [21-44], four were included in the meta-analysis for the SOFA (Figure 7) [26,36,38,39]. After conducting a random effects model, a moderate estimate of effect size (Cohen’s d value=1.40, 95% CI: 0.70 to 2.10, p<0.001) was revealed, indicating that the SOFA was significantly different in the group of survivors (controls) compared to the non-survivors group (deceased). Significant heterogeneity was identified across included studies (p<0.001, I^2^=99.3%). Visual examination of funnel plots and Egger's test (p=0.697) indicated that no significant publication bias was revealed over all included studies. For these four studies the moderate estimate of effect size of TRISS was lower than before (Cohen’s d value = -1.71, 95% CI: -1.99 to -1.43, p<0.001) [26,36,38,39].

Μeta-Regression Analysis

A random-effects meta-regression analysis was conducted using the Restricted Maximum Likelihood (REML) estimator in order to examine whether study design (retrospective versus prospective) and year of the study predicted the effect sizes of the study. The overall year-only model was not statistically significant, Wald χ^2^(1)=0.069, p=0.793, indicating that year did not significantly explain variation in effect sizes. Additionally, the overall model for the study design (retrospective versus prospective) and the year of the study was non-significant, Wald χ^2^(2)=0.150, p=0.928, indicating that the combination of these two moderators did not significantly explain the variation in effect sizes. The R^2^ in both meta-regression models, which was 0%, indicated that the included moderators did not explain additional variance in effect sizes.

Moreover, a sensitivity analysis was performed by restricting the analysis to the most recent studies (published after 2015). The pooled effect estimate remained stable, with a non-significant result (p=0.982), indicating that the overall findings were robust and not driven by older studies.

Discussion

In this systematic review and meta-analysis, we evaluated the prognostic performance of the Trauma and Injury Severity Score (TRISS) in comparison to established ICU scoring systems, including APACHE II, APACHE III, and SOFA, among adult polytrauma patients admitted to intensive care units. TRISS demonstrated a strong ability to discriminate between survivors and non-survivors, with a pooled effect size of Cohen’s d = -1.54 (95% CI: -1.73 to -1.35, p<0.001). Subgroup analyses revealed that in the 12 studies used for APACHE II comparison, the effect size for TRISS (Cohen’s d = -1.55) was similar in magnitude to that of APACHE II (Cohen’s d=1.53) [21,22,26,28,30,32,36-38,40,42,43]. In the three studies analyzed for APACHE III, TRISS had an effect size of -1.20, compared with 1.53 for APACHE III [23,25,37]. In the four studies analyzed for SOFA, TRISS demonstrated an effect size of -1.71, compared with 1.40 for SOFA [26,36,38,39]. Despite considerable heterogeneity among studies (I^2^>99%), no significant publication bias was detected. These findings validate the enduring clinical relevance of TRISS as a prognostic tool in the management of trauma patients in the ICU.

The TRISS score demonstrated moderate to excellent discriminatory ability across the included studies in ICU patients with multiple trauma, with AUROC values typically ranging from 0.64 to 0.96 [21-26,28-41,43,44]. Similar results were found in three other studies using the TRISS scale in multiple trauma patients requiring ICU admission, with mean ISS values around 20, indicating moderate to severe trauma severity [8,45,46]. At the same time, Orhon et al. showed that this scale can predict the need for mechanical ventilation and also predict mortality [47].

The systematic review highlighted some of the weaknesses in the TRISS scale across certain subgroups of patients. According to Magee et al., it showed a reduced discriminatory power, with an AUROC value of 0.64 [25], a fact confirmed by Reiter et al., who observed that it tends to underestimate mortality in patients over 60 years of age [31]. This age group is a high-risk population, as pre-existing comorbidities such as cardiovascular disease and chronic respiratory illness are recognized as critical aggravating factors for their overall prognosis [48,49]. The high mortality rate of older trauma patients suggests the need for different treatment strategies [48]. On the contrary, Chico-Fernández et al., who studied very elderly multi-injured patients (over 80 years) hospitalized in an ICU, found that the actual outcome of the patients was better than that predicted by the Injury Severity Scores [50]. It is worth noting that Jiang et al. found the accuracy of TRISS to be higher than the APACHE II and SAPS II in predicting in-hospital mortality among geriatric trauma patients [51]. Consistent with these findings, the meta-analysis by Liu et al. demonstrated that TRISS has better accuracy and performance in predicting mortality among trauma patients over 60 years old than the ISS and Geriatric Trauma Outcome Score (GTOS) [52].

Similarly, in terms of wound mechanism, TRISS demonstrated better predictive ability for penetrating trauma compared with blunt trauma [33]. More specifically, in a multicenter study, TRISS showed better calibration for penetrating trauma, with a Hosmer-Lemeshow test score of 5.91 (p=0.658), compared with blunt trauma, which had a score of 27.35 (p<0.0001) [33]. The ISS scale, although widely used, has inherent limitations in capturing the complexity of severe injuries, leading to an underestimation of risk in multi-injured patients. Meanwhile, the NISS outperforms the ISS in predicting the outcomes for the severe blunt trauma scale as it assesses all severe injuries regardless of site, leading to a more realistic depiction of the patient's overall burden [53].

This systematic review and meta-analysis also compared TRISS with other prognostic tools used in the ICU. The TRISS model demonstrated prognostic accuracy comparable to APACHE II and APACHE III, and it slightly outperformed the SOFA score. In a similar meta-analysis Yu et al. compared APACHE II with TRISS in 4,054 multitrauma patients and showed that both scales could accurately predict mortality in this category of patients in the ICU [12]. As for the APACHE III scale, despite having the same ability as TRISS to predict outcome [23,25,37], it is noteworthy that the practicality and usability of TRISS results from the requirement for less and more readily available data, as well as its focus on wound characteristics, without the need to collect extensive parameters within 24 h in the ICU setting [23]. However, in several comparative studies, the SOFA score is often preferred due to its ease of calculation and its usefulness in monitoring the progression of organ dysfunction in critically ill patients [26,36,38,39]. Studies such as by Niaz et al. and Antonelli et al. have supported the value of the SOFA scale for both prognosis and monitoring the condition of these patients [54,55].

An important finding of this study is that the addition of other clinical variables to the TRISS scale, such as heart rate variability indices [44], base deficit measurements [24,35], or other clinical scores, has emerged as a strategy to improve the predictive accuracy of the scale [31,39]. In a multicenter study by Domingues et al., adaptation of the TRISS scale was performed, where these new models showed good accuracy and similar performance to the original TRISS [10]. It is worth noting that the TRISS methodology has been validated and refined over time; however, these variants were not used entirely in ICU patients [9,56,57]. This suggests that while TRISS provides a robust anatomical and physiological foundation [8,15], its adaptability to the complex ICU environment may be strengthened by incorporating dynamic markers of physiological reserve and organ dysfunction, which are not captured in the original method [58]. These combined approaches enhance the discriminative ability of the TRISS in multitrauma patients admitted to the ICU, which is an environment with a complex clinical picture.

This review presents a number of limitations that need to be considered. Initially, the literature search was restricted to studies published in English, which may have introduced language bias and led to the exclusion of potentially valuable studies published in other languages. In addition, significant heterogeneity was found among the studies included in the meta-analysis (I^2^>99%), which may be due to differences in population characteristics, such as variation in wound severity (different ISS values), age distribution of patients, methods of calculating prognostic indicators, and differences in treatment protocols used in ICUs. Furthermore, the evolution of the Abbreviated Injury Scale (AIS) over time is a potential confounding factor. As the AIS has undergone several revisions, the studies included in our analysis span a period where different versions were in use [5]. Although the core principles remain consistent, subtle changes in coding and injury severity classifications across versions could introduce heterogeneity in the calculation of the ISS and, consequently, the TRISS score, which may affect the pooled accuracy estimates [59]. Also, some of the studies included in the meta-analysis were retrospective, which may have affected the data's accuracy due to an increased risk of errors in recording and measuring predictor variables (information bias). Finally, even though we explored heterogeneity with meta-regression and sensitivity analyses, additional approaches such as leave-one-out analysis were not feasible due to the limited number of studies in some prognostic score subgroups. Future research, including a larger number of studies and more homogeneous datasets, may allow the application of these methods to provide deeper insights.

## Conclusions

In conclusion, the present systematic review and meta-analysis highlighted TRISS as a useful and reliable prognostic tool for assessing outcome in multitrauma patients admitted to the ICU. The findings indicate that TRISS has predictive ability comparable to that of widely used ICU scores, such as APACHE II, APACHE III, and SOFA, while retaining the advantage of simpler, faster implementation using readily available clinical data. To further improve the functionality and accuracy of TRISS in the ICU setting, it is proposed to include additional clinical variables. The addition of these parameters may increase its adaptability to the specific requirements of intensive care and enhance its predictive accuracy in multi-injured patients.

## Appendices

**Table 3:** Detailed database search strategies. Full electronic search strategies for PubMed, ScienceDirect, Scopus, and CINAHL. Search terms combined using Boolean operators (AND/OR).

Databases	Search terms	
PubMed	#1	("TRISS"[tiab] OR "Trauma and Injury Severity Score"[tiab])
	#2	#1 AND ("Intensive Care Units"[MeSH] OR "ICU"[tiab] OR "Critical Care"[tiab])
	#3	#2 AND ("Multiple Trauma"[MeSH] OR "Trauma"[tiab] OR "Severe Injury"[tiab])
	#4	#3 AND ("APACHE II"[tiab] OR "APACHE III"[tiab] OR "SOFA"[tiab])
ScienceDirect	#1	("TRISS" OR "Trauma and Injury Severity Score")
	#2	#1 AND ("Intensive Care Units" OR "ICU" OR "Critical Care")
	#3	#2 AND ("Multiple Trauma" OR "Trauma" OR "Severe Injury")
	#4	#3 AND ("APACHE II" OR "APACHE III" OR "SOFA")
Scopus	#1	TITLE-ABS-KEY("TRISS" OR "Trauma and Injury Severity Score")
	#2	#1 AND TITLE-ABS-KEY("Intensive Care Units" OR "ICU" OR "Critical Care")
	#3	#2 AND TITLE-ABS-KEY("Multiple Trauma" OR "Trauma" OR "Severe Injury")
	#4	#3 AND TITLE-ABS-KEY("APACHE II" OR "APACHE III" OR "SOFA")
CINAHL	#1	("TRISS" OR "Trauma and Injury Severity Score")
	#2	#1 ("Intensive Care Units" OR "ICU" OR "Critical Care")
	#3	#2 ("Multiple Trauma" OR "Trauma" OR "Severe Injury")
	#4	#3 ("APACHE II" OR "APACHE III" OR "SOFA")
